# The Technical Quality of Delivered Care for People with Inflammatory Bowel Disease in Tabriz Gastroenterology Clinics

**DOI:** 10.15171/hpp.2015.024

**Published:** 2015-10-25

**Authors:** Jafar Sadegh Tabrizi, Mohammad Hossein Somi, Sima Asghari, Mohammad Asghari Jafarabadi, Farid Gharibi, Saeideh Alidoost

**Affiliations:** ^1^Tabriz Health Services Management Research Center (NPMC), Tabriz University of Medical Sciences, Tabriz, Iran; ^2^Gastrointestinal and Liver Disease Research Center, Tabriz University of Medical Sciences, Tabriz, Iran; ^3^Department of Health Services Management, Tabriz University of Medical Sciences, Tabriz, Iran; ^4^Road Traffic Injury Research Center, Tabriz University of Medical Sciences, Tabriz, Iran; ^5^Student’s Research Committee, Tabriz University of Medical Sciences, Tabriz, Iran; ^6^Student’s Research Committee, Department of Health Services Management, Tabriz University of Medical Sciences, Tabriz, Iran

**Keywords:** Inflammatory Bowel Disease, (IBD), Technical Quality, Diet

## Abstract

**Background: ** The Inflammatory Bowel Disease (IBD) is considered as one of the chronic diseasesre-quiring complicated treatment. This study aimed to assess technical quality of providing care for pa-tients with IBD.

**Methods:** This cross-sectional study was conducted on 94 people with IBD using interviews and simple random sampling methods in Gastroenterology, Endoscopy and clinic of Imam Reza Hospital and Golgasht Clinic in Tabriz in 2012. The data collection tool was a researcher-designed questionnaire whose validity and reliability had been confirmed. In order to investigate the statistical relationship between the background variables and compliance with the standards the Chi-square test was applied using SPSS 17 Software.

**Results:** "visit by the physician" and "diet advice by the dietitian" have had the highest and the lowest levels of compliance with the standard respectively, and "the care related to the disease exacerbation" and "the care provided by the other physicians" were not compatible with the standards in 80% of the cases. Data analyses also showed that there was a significant relationship between participant’s age, job, education and the smoking status and compliance of some care with the relevant standards (P<0.05).

**Conclusion:** The results indicate a substantial gap between provided care for the people with IBD and the relevant standards. This indicates the areas that need of improvement and requires the serious attention of the authorities.

## Introduction

IBD refers to a set of chronic inflammatory bowel conditions with unknown causes and two of its major diseases are Crohn’s disease (CD) and Ulcerative colitis (UC).^1^ The incidence and prevalence of IBD in Asia is less than Western countries.^2^ So, a significant growing trend is evident in the Asian countries including China, South Korea, India and Iran, that have adopted to the western lifestyle.^2^ The incidence of UC is more than CD in Iran and the incidence of IBD is more frequent in youth and among men.^3^

The treatment of IBD is complex due to the existence of different alternatives for treatment, and, the gradual and continuous evolution of the physicians' perception of the risks and advantages of different treatments. Consequently, standardization of the provided care for IBD is difficult. Much of IBD care is provided without using IBD guidelines. Differences in care provision shows the low quality of care and reveals overuse, underuse and misuse of resources evident for CD and UC diseases.^4^

Given the nature of IBD and its considerable effects on people's life and the considerable burden of IBD costs for patients, families, society and health system, attention to the quality of provided care and its improvement seems necessary.^5^ According to the definition of the Medical Institute of America, quality of health care refers to levels of health services provided for people and societies which increase the possibility of optimal health outcomes and are in accordance with the modern professional knowledge.^6^

Different approaches are used to evaluate the quality of health care.^7,8^ In a comprehensive model entitled "Comprehensive Quality Measurement in Health care(CQMH)", quality of health care was assessed in the three dimensions of service quality, technical quality and customer quality.^9^ Technical quality mostly reflects knowledge, skills and capabilities of the service provider and refers to what customer received rather than what has been introduced as the service standards based on scientific evidence.^10^

In order to evaluate provided care based on standards and guidelines, different resources including paper medical records, electronic health record systems, administrative data, registries, stimulation, observation and customers' report are used.^5^ In CQMH model, in order to investigate technical quality, the provided care is evaluated based on guidelines and standards from the customers' viewpoint (recipients of services). The health care customer is one of the important resources in evaluation of quality providing valuable and unique data.^10^

Thus, the present study was aimed to assess technical quality of provided care for people with IBD based on IBD care standards from the customer’s viewpoint.

## Materials and Methods

This cross-sectional study was conducted in Gastroenterology clinics of Imam Reza Hospital and Golgasht in Tabriz in 2012. The desired sample size of 94 was calculated using the formula (n=z^2^s^2^/d^2^) with estimates of the mean and standard deviations of the total scores of technical quality estimated to be 78 and 38.5, respectively, based on a preliminary study including 30 people and accepting confidence level of 95% and d^2^=0.1. The sampling method was conducted using a simple random sampling and the inclusion criteria consisted of subject shaving medical records in the investigated area, constant visiting of the physician over a year and patient willingness to take part in the study. Patients were excluded if they did not wish to participate in the study or if they had a severe IBD or disease exacerbation at the time of the study.

The data collection tool was a questionnaire with 2 sections: personal information and technical quality (including 28 questions). Two questions related to the general status of disease control according to the patient and disease exacerbation and 26 questions related to the care standards.

Study questions were developed based on the opinion of 10 experts related to important areas of care. The content validity of the questionnaire was confirmed using Content Validity Index (CVI) and the Content Validity Ratio (CVR) tested with 9 experts.^11,12^ In order to calculate CVI, three standards of relevance, clarity and simplicity were used on a 4-pointLikertscale. So, CVI of each question was calculated by dividing the number of agreeing experts (persons with ranks 3 and 4) by the total number of the experts (separately for each of the three standards). In order to determine CVR, the experts were asked to examine each question based on the 3-point scale ("it is necessary", "it is useful and unnecessary" and "it is not necessary"). Subsequently, the answers were calculated based on the following formula: QUOTE

In the above formula, nE is the number of experts that have rated for the "it is necessary" alternative and N is the total number of experts. If the calculated CVR would be larger than the CVR table the content, validity was confirmed. In addition, the reliability of the questionnaire was confirmed by the Cronbach's alpha equal to 0.79. The CVI was 0.89 and the CVR was 0.8 indicating a high level of expert agreed validity.

Study participants reported the level of services received and then the reported level was compared to the care standards to show the degree of adherence to care in terms of frequency (percentage). Data were presented by frequency (percent) to investigate the statistical relationship between background variables and compliance with the care standards and the characteristics related to the disease conditions (disease control and disease exacerbation).Chi-square test was used based on the exact method.^13,14^ Data was analyzed using the SPSS 17 Software (Chicago, IL, USA) and *P*<0.05 was considered as significant.

### Ethical Considerations

Ethical principles considered in this study included the complete freedom of all participants to accept or refuse to cooperate in the study, obtaining informed consent from participants, respecting the privacy of participants and ensuring the participants in that the use of their data and information was exclusively in line with the study goals. In addition, this study was approved by Ethics Committee of Tabriz University of Medical Sciences.

## Results

In the present study, the participation rates of women and men were almost equal and most of the responders were native. Most of the participants had a normal weight, were employed covered by one of the health insurances. Only, 14% of participants were current or past smokers. In addition, most of the participants had finished high school or lower level of education (Table 1).

**Table 1:** Demographic information of the participants

	**Demographic features**	**Frequency**	**Percentage**
Gender	Male	48	51.1
	Female	46	48.9
Age (yr)	27≤	32	34.0
	28-40	32	34.0
	41≥	30	31.9
Nativity status	Native	55	58.5
	Non-native	39	35.5
BMI index	18.5≤	4	4.3
	18.51-25	47	50.0
	25/01-30	35	37.2
	30>	8	8.5
Smoking status	Smoking	3	3.2
	Non-smoking	91	96.8
Smoking records	Yes	13	13.8
	No	81	86.2
Job	Employed	42	44.7
	Housewife/husband	25	26.6
	Unemployed	8	8.5
	Student	14	14.9
	Retired	5	5.3
Level of education	High school or lower	59	62.7
	University education	35	37.3
Insurance status	Yes	91	96.8
	No	3	3.2

The disease control status has been optimal for most of participants (56%) and more than 55% have had disease exacerbation over the past 12 months (Table 2).

**Table 2:** The features related to the disease conditions

**Component**		**Frequency**	**Percentage**
The disease control status according to the patient	Poor	11	11.8
	Average	30	32.3
	Optimal	52	55.9
Disease exacerbation	Yes	54	57.4
	No	40	42.6

Table 3 shows the level of compliance of the care provided with the standards in the form of three sets of care including "clinical tests and care provided at each visit", "the care provided in case of disease exacerbation" and "the care provided by other professionals". The findings indicate that "training the smokers regarding the role of smoking and its relationship with IBD" is the only care in which the level of compliance with the standards is 100%.

**Table 3:** The status of compliance of the care provided for the patients suffering from IBD and the standards

	**Type of care**	**Care standards over 12 months**	**Comparison with the standards**	
			**Frequency (percentage) of non-compliance with the standards**	**Frequency (percentage) of compliance with the standards**
**Clinical tests and care provided in each visit**	Visit by the physician	3 time and more	12 (12.9)	81 (87.1)
	The patient being questioned regarding the consumption of pain relief medicine (NSAIDs) by the physician	3 time and more	66 (70.2)	28 (29.8)
	The patient being questioned regarding diarrhea, its time and times by the physicians	3 time and more	21 (22.6)	72 (77.4)
	The patient being questioned regarding the relationship between the severity of symptoms and the type of food by the physician	3 time and more	53 (57.6)	39 (42.4)
	The patient being questioned regarding the relationship between diarrhea and the quality of wakefulness and sleep by the physicians	3 time and more	63 (68.5)	29 (31.5)
	The patient being questioned regarding the blood in the stool (rectal bleeding) by the physician	3 time and more	16 (17.2)	77 (82.8)
	The patient being questioned regarding the disposal of medications through stool by the physicians	3 time and more	62 (66.7)	31 (31.3)
	Examination of the current diet by the physician	3 time and more	70 (74.5)	24 (25.5)
	The patient being questioned regarding the drug tolerance and the medication time by the physician	3 time and more	26 (28.0)	67 (72.0)
	The patient being questioned regarding physical/economic access to prescribed medicine by the physician	3 time and more	77 (83.7)	15 (16.3)
	Training the smokers regarding the role of smoking and its relationship with IBD	3 time and more	0 (0.0)	93 (100)
	Number of stool tests	Once	48 (51.6)	45 (48.4)
	Number of blood tests	Twice	36 (38.3)	54 (61.7)
**The care provided in case of disease exacerbation**	The patient being questioned regarding the psychological state (in case of disease exacerbation)	3 time and more	48 (88.9)	6 (11.1)
	The patient being questioned regarding the drug tolerance and medication time (in case of disease exacerbation)	3 time and more	34 (63.0)	20 (37.0)
	The patient being questioned regarding the disposal of medication through stool (in case of disease exacerbation)	3 time and more	43 (79.6)	11 (20.4)
	The patient being questioned regarding access to medicine (in case of disease exacerbation)	3 time and more	52 (96.3)	2 (3.7)
	The patient being questioned regarding the pain relief medicine (NSAIDs) (in case of disease exacerbation)	3 time and more	48 (88.9)	6 (11.1)
	Investigation of the diet (in case of disease exacerbation)	3 time and more	46 (85.2)	8 (14.8)
	Special diet advice (in case of disease exacerbation)	3 time and more	53 (98.1)	1 (1.9)
**The care provided by the others**	Visiting the dietitian	Once	86 (93.5)	6 (6.5)
	Diet advice by the dietitian	Once	89 (95.7)	4 (4.3)
	Examination by the ophthalmologist	Once	69 (73.4)	25 (26.6)
	Examination of joints by the professional	Once	83 (88.3)	11 (11.7)
	Examination of skin by the professional	Once	81 (86.2)	13 (13.8)
	Examination of osteoporosis (densitometry)	Once	75 (79.8)	19 (20.2)

The level of compliance with the standards is more in the first group (common measures) compared with the second group (the care related to the disease exacerbation) and the third group (the care provided by the other professionals). In the set of the first group care, "visits by the physician", "the patient being questioned regarding blood in the stool (rectal bleeding) by the physician", " the patient being questioned regarding the drug tolerance and the medication time by the physician" and "the patient being questioned regarding diarrhea, its time and times by the physician" are compatible with the standards in more than 75% of the cases on average and in other cases the level of compliance with the standards is very low. In the set of care related to the disease exacerbation and the care provided by other professionals, also compliance with the standards has been at a very low level and the average compliance with the standards is 20% and 10% respectively.

Investigation of the statistical relationship between the disease control status according to the participants and their demographic features showed that there was a relationship between the disease control status and gender (*P*=0.021) and BMI (*P*=0.026) such that compared with men, women have evaluated their disease control status as optimal or average and "the poor disease control" status has been more among men. In addition, the disease control has had a more optimal status among persons with normal weight and overweight compared with thin and heavy people (Table 4).

**Table 4:** Relationship between disease control status and demographic variables

**Demographic features**		**Disease control status**			**P-value**
		**Poor**	**Average**	**Strong**	
**Gender**	Female	2 (4.3)	16 (34.8)	28 (60.9)	0.021
	Male	10 (20.9)	14 (29.1)	24 (50.0)	
**Age**	27≤	8 (25.0)	8 (25.0)	16 (50.0)	0.274
	28-40	2 (6.3)	12 (37.5)	18 (56.2)	
	41≥	2 (6.7)	10 (33.3)	18 (60.0)	
**Native**	Native	7 (12.7)	17 (31.0)	31 (56.3)	0.917
	Non-native	5 (12.8)	13 (33.3)	21 (53.9)	
**BMI**	18.5≤	0 (0.0)	0 (0.0)	4 (100)	0.026
	18.51-25	6 (12.8)	12 (25.5)	29 (61.7)	
	25.01-30	4 (11.4)	12 (34.3)	19 (54.3)	
	>30	2 (25.0)	6 (75.0)	0 (0.0)	
**Job**	Employed	2 (4.7)	17 (40.5)	23 (54.8)	0.337
	Housewife/husband	4 (16.0)	5 (20.0)	16 (64.0)	
	Unemployed	1 (12.5)	2 (25.0)	5 (62.5)	
	Student	4 (28.6)	4 (28.6)	6 (42.8)	
	Retired	0 (0.0)	2 (40.0)	3 (60.0)	
**Education**	Diploma or lower	7 (11.8)	15 (25.5)	37 (62.7)	0.222
	Academic education	4 (11.4)	15 (42.9)	16 (45.7)	
**Insurance**	Yes	11 (12.1)	29 (31.8)	51 (56.1)	0.409
	No	0 (0.0)	2 (66.7)	3 (33.3)	

The findings of Table 5 indicate that there is a significant relationship between "investigation of drug tolerance and medication time by the physician" and the age group (*P*=0.028) and job (*P*=0.022); such that among employed people and the age group of above 41 yr the level of compliance with the standards was higher. Moreover, there is a statistical relationship between "investigation of physical/economic access to medicine by the physician" and smoking (*P*=0.016). Smoking (*P*=0.041) and smoking records (*P*=0.036) have a relationship with "investigation of the patient's psychological state in case of disease exacerbation" such that among non-smokers and persons with no smoking records investigation of the psychological state has had a higher compliance with the standards. There is a significant relationship between "Examination by the ophthalmologist" and education (*P*=0.006) and job (*P*=0.037) and also between compliance with the standard of "investigation of osteoporosis" and the nativity status of the individuals; such that the level of visits to the ophthalmologist among the persons with university education and for employed persons and "investigation of osteoporosis" among natives (*P*=0.011) have been more compared with the other persons (Table 5).

**Table 5:** The statistical relationship between adherence to care standards and demographic variables

**Demographic features**		**The status of compliance of the care provided with the standards**		**P-value**
**Investigation of drug tolerance and medication time by the physician**				
		Below standard	Standard and higher	
**Age group**	≤27	12 (37.5)	20 (62.5)	0.028
	28-40	11 (35.5)	20 (64.5)	
	≥41	3 (10.0)	27 (90.0)	
**Job**	Employed	9 (22.0)	32 (78.0)	0.022
	Housewife/husband	7 (28.0)	18 (72.0)	
	Unemployed	6 (75.0)	2 (25.0)	
	Student	4 (28.6)	10 (71.4)	
	Retired	0 (0.0)	5 (100)	
**Investigation of physical/economic access to medicine by the physician**				
**Smoking status**	Smoking	1 (33.3)	2 (66.7)	0.016
	Non-smoking	76 (85.4)	13 (14.6)	
**Investigation of psychological state in case of disease exacerbation**				
**Smoking records**	Yes	4 (30.8)	9 (69.2)	0.036
	No	50 (62.0)	31 (38.0)	
**Smoking status**	Smoking	0 (0.0)	3 (100)	0.041
	Non-smoking	54 (59.3)	37 (40.7)	
**Examination by the ophthalmologist**				
**Education**	Diploma or lower	49 (77.8)	14 (22.2)	0.006
	Academic education	21 (87.5)	3 (12.5)	
**Job**	Employed	28 (84.8)	5 (15.2)	0.037
	Housewife/husband	22 (91.7)	2 (8.3)	
	Unemployed	3 (75.0)	1 (25.0)	
	Student	12 (42.9)	16 (57.1)	
	Retired	4 (57.1)	3 (42.9)	
**Investigation of osteoporosis**				
**Native**	Native	39 (71.0)	16 (29.0)	0.011
	Non-native	36 (92.3)	3 (7.7)	

## Discussion

This study aimed to assess technical quality of delivered care for people with IBD from the patient’s viewpoint. The study indicate that the highest level of compliance with the standards is related to "the visits by the physician" such that more than 87% of the patient reported the number of visits to the physician to be according to the standard. Most of the care provision including examination of pain relief medicine, investigation of the relationship between diarrhea and the quality of wakefulness and sleep, investigation of disposal of medications by stool, investigation of diet and investigation of the patient's physical/economic access to medicine that must be provided at each visit according to standard, were not consistent with the standards. Nevertheless, in the study by Sadlo et al. more than 50% of the patients have expressed satisfaction with access to the prescribed medicine and the providers' attention to this issue.^15^

In this study, although the number of visits conducted by the physicians is more consistent with the standards, most of the care is not provided at regular times based on the standard. Different evidence also indicates that evaluation of the patients suffering from IBD is inadequate and that there is a substantial gap between the standards and the care provided for the patients.^1,16,17^

As management of IBD is a complex and dynamic process depending on disease status, and, in case of the disease exacerbation, treatment of patients and their potential effects requires special attention and practice based on the treatment guidelines.^5^ The results of the present study show that more than 57% of the patients have had disease exacerbation over a year and on average the reports of more than 85% of these patients indicates non-compliance with the relevant standards.

The level of compliance of "investigation of the patients' psychological state in case of disease exacerbation" with the related standard wasvery low and performed only for 10% of the patients.This is consistent with the findings of others. In the study by Evertsz et al. in which more than 40% of the patients suffering from IBD have been suffering from one of the mental disorders, only 18% of the patients had received psychiatric and psychological services.The psychological services provided for the patients with optimal mental state werevery low as well which indicates inadequate attention to prevention from mental disorders in the patients.^18^ However, the study by Sadlo et al. showed different results with a much higher level of appropriate investigation of the psychological state of the patients suffering from IBD and effective treatment of more than 65% of the psychological or psychiatric patients.^15^

IBD can involve eyes, skin and other parts of the body such as the finger joints, hands, legs and the spine, and appropriate care requires the involvement of relevant other professionals. In the study by Sadlo et al.^15^ more than 76% of the patients suffering from IBD reported being satisfied with participation of other professionals; yet in the present study most patients report non-compliance of provision of care related to the other expertise including examination by the ophthalmologist, examination of the joints, examination of the skin, visits to the dietitian, the diet advice by the dietitian and investigation of the osteoporosis with the standards.

Although special diets are not effective in the treatment of IBD in the long term, using a diet in accordance with the disease state will help to the improvement of the symptoms and better function of the medicines.^19^ However, in the current study there is a substantial gap between the care related to nutrition including "investigation of diet by the professional, visits to the dietitian and special diet advice in normal conditions and the disease exacerbation" and the relevant standards.

Individual training of patients leads to an increase in their knowledge regarding the disease and consequently compliance with medical treatments, increase in the patients' satisfaction and reduction in frequent visits.^20^ In the present study, investigation of smoking status and counselling the smokers had been performed in 100% of the cases. However, there are ambiguities concerning the effects of smoking in IBD such that some studies report aprotective effects of smoking in IBD.^21^

The research findings show that employment status is related to two standards, “investigation of drug tolerance and medication time by the physician" and "examination by the ophthalmologist" with a higher compliance with the standards among the employed persons compared with others. In addition, there is a significant relationship between age and "investigation of drug tolerance and medication time by the physician" such that amongolder participants the level of compliance with the standards. This may be due to is higher possibly higher necessity of provision of this care among this group persons or high expectations due to greater knowledge (and consequently request for standard care). There is also a significant relationship between the level of education and "examination by the ophthalmologist" such that compliance with the standards in the persons with a higher level of education is higher. Again, this may be due to higher knowledge and awareness of the personsof the need for regular ophthalmologist assessment.

Pallis et al. in Greece reported significant relationship between age, education and duration of illness and satisfaction of the patients suffering from IBD regarding the provided care.^22^

Regarding the limitations of the present study, as there were no approved guidelines for the care provided for the patients suffering from IBD, the expert opinion of the relevant professionals was used to establish the study IBD care standards. In addition, this study was conducted from the viewpoint of patients suffering from IBD (customers' reports) and there is the possibility of recall and information bias.

## Conclusion

There is a substantial gap between the IBD care standards and provided care for people with IBD, and this gap is more evident in the care related to the disease exacerbation and the care provided by the other professionals. This shows a lack of awareness or inattention of the providers regarding IBD standards and very low attention to the importance of a multidisciplinary management team for IBD treatment.

## Acknowledgements

The researchers hereby thank all the staff of the Departments of Gastroenterology, Endoscopy and the clinic of Imam Reza Hospital of Tabriz and Golgasht Clinic of Tabriz and the patients participated in the study. We appreciate the Student Research Committee of Tabriz University of Medical Sciences for their financial support of the project.

## Competing Interests

The authors declare that there is no conflict of interest.
